# Research on Artificial Intelligence Classification and Statistical Methods of Financial Data in Smart Cities

**DOI:** 10.1155/2022/9965427

**Published:** 2022-01-06

**Authors:** Xuezhong Fu

**Affiliations:** School of Economics, Hainan University, Haikou 570228, China

## Abstract

In order to improve the effect of financial data classification and extract effective information from financial data, this paper improves the data mining algorithm, uses linear combination of principal components to represent missing variables, and performs dimensionality reduction processing on multidimensional data. In order to achieve the standardization of sample data, this paper standardizes the data and combines statistical methods to build an intelligent financial data processing model. In addition, starting from the actual situation, this paper proposes the artificial intelligence classification and statistical methods of financial data in smart cities and designs data simulation experiments to conduct experimental analysis on the methods proposed in this paper. From the experimental results, the artificial intelligence classification and statistical method of financial data in smart cities proposed in this paper can play an important role in the statistical analysis of financial data.

## 1. Introduction

With the acceleration of economic globalization, financial information has become more transparent and authentic. In this context, accounting standards are converging faster and faster globally. According to incomplete statistics, there are nearly 120 countries and regions that have adopted the International Financial Reporting Standards (IFRS) in the global plan or have adopted the International Financial Reporting Standards (IFRS). The international financial crisis that broke out in 2008 made people realize that improving the transparency of accounting information and formulating a set of globally unified and high-quality accounting standards are vital to the stability and healthy development of the global financial system and capital markets. In this context, countries and regions have accelerated discussions and research on the international convergence of accounting standards [1].

Because keyword retrieval technology cannot meet the needs of some occasions, in recent years, some new researches have begun to focus on improving the efficiency and accuracy of information retrieval technology. Information extraction technology is one of the technologies for obtaining specific events or the relationship between events and events. It is a process of extracting structured and unambiguous information from unstructured free text or other information resources. Information extraction technology can not only filter out information that is not useful to users, but also generate specific information that users are interested in. Information extraction technology integrates artificial intelligence and natural language processing technology and plays an indispensable role in the field of information retrieval. To evaluate the market value of a company, such as the evaluation of intangible assets, all financial data of the company and the financial data of the industry are required, including the company's financial statements and notes to the statements, relevant industry financial news, and macro- and microeconomic data [2]. Most of these data are stored on the Internet in formats such as web pages (HTML) and PDF and are distributed on various websites that provide financial information. At present, people can only manually collect and sort relevant financial data from different data sources, carefully sort and analyze and filter out the data they want, but there is no place to directly get all the data they want. This takes a lot of manpower and time, and most of the work is repetitive work. According to the preestablished evaluation model, the financial data evaluation results are obtained, which will greatly improve the efficiency of decision-making and provide support for financial-related decisions such as stock investment, mergers and acquisitions, and financial risk assessment [3].

This article combines big data technology to conduct financial data classification research and explores intelligent methods suitable for contemporary financial data classification.

## 2. Related Work

The literature [4] put forward some suggestions on the classification of financial assets. It includes two parts: one is the understanding of the different levels of financial transactions, and the other is how to deal with the various levels of relationships in the classification of financial instruments during the transaction process. The literature [5] conducted a comparative analysis on the four categories of financial assets and believed that financial assets should also be included in the foreclosed assets, and the financial assets in this part should be handled in accordance with the relevant regulations in the four categories. The literature [6] specifically analyzed and explained the revision of the classification of nonfinancial assets and financial assets in the national economic accounting system. The literature [7] studied the classification of financial instruments and the measurement model of financial instruments, explored the standard and root causes of the classification of financial instruments, and studied the future direction of the classification standard. According to the current classification standards of financial instruments, the literature [8] elaborated the classification standards of held-to-maturity investments in detail. The literature [9] believed that, under the current standards, the main reason for the strong subjectivity of the classification of financial assets lies in the intention of the management. Therefore, there may be errors in the classification of financial assets in actual work, and the relevant discrimination methods are given in the article.

With the changes in the economic environment, follow-up measurement issues and other related issues derived from the classification of financial assets have gradually emerged, and scholars have launched relevant discussions on these issues. Since the promulgation of the new accounting standards and the full implementation of the financial instrument standards, many scholars have begun to pay attention to whether the problem of financial asset classification will bring earnings management opportunities to enterprises. Therefore, most of the research and discussion on this issue have been concentrated in recent years. Literature [10] believes that the current research does not clearly define the criteria for the classification of financial assets, especially for transactional financial assets and available-for-sale financial assets, which only require classification according to the holding intention and ability of the enterprise. Therefore, companies can make full use of the subjectivity of financial asset classification to manipulate corporate profits. Literature [11] made a specific analysis on the impact of the classification of equity financial assets on the basis of a full analysis of corporate equity financial assets. Literature [12] conducted an empirical study on the classification of listed companies' financial assets under the new standards. The research perspective is management, accounting policies, and earnings management. Studies have shown that when the initial classification of financial assets is performed, listed companies have obvious intentions to hold financial assets and can be clearly classified; however, during the holding period of financial assets, listed companies have more room for profit operation. The accounting treatment of available-for-sale financial assets can be managed according to the management's wishes. Literature [13] analyzed the classification standards of financial instruments of listed companies and concluded that because the classification of financial instruments is subjective and selective, holders will choose according to their own interests to maximize the reliability of financial information. Literature [14] investigated and studied the classification of financial instruments of listed companies and concluded that the classification of financial instruments of listed companies has a certain tendency according to the wishes of management. It can be found that many scholars have discovered in their research that the classification of financial assets will indeed bring opportunities for companies to manage earnings. The classification of financial assets is closely related to fair value measurement, and some scholars have discussed this. Literature [15] puts forward new insights in the classification, reclassification, and measurement of financial assets to prevent corporate management from adjusting corporate profits at will. Research in this area also exists in the field of practice. Literature [16], in studying the classification of financial instruments of securities companies and the fair value of financial instruments, concluded that the accounting information disclosure system of enterprises on financial instruments is still not perfect. Commercial banks hold the largest scale of financial assets, so some scholars have carried out research on the impact of financial asset classification on commercial banks' profits. Literature [17] analyzed the classification of financial instruments of commercial banks and concluded that the management of the enterprise has a risk appetite for the classification of financial instruments, and it replaces liquidity with robustness. Literature [18] uses the data of Liangmianzhen, Minmetals Development, and Qianjiang Biochemical Company as examples to discuss the earnings management of listed companies based on the perspective of financial asset classification and accounting confirmation differences. Literature [19] compares the differences between IFRS9 and the current accounting standards in the classification of financial assets and analyzes the differences between IAS39 and IFRS9 financial asset classification of 16 listed banks, showing the possible impact of commercial banks in the future. Financial asset standards are closely related to the financial environment. Literature [20] puts forward suggestions for strengthening supervision and protection. Literature [21] discusses the problems that financial asset reclassification may bring to corporate profits and losses, including the recognition of corporate profits and losses in financial asset holdings, the impact of financial asset impairment on profits and losses, and the possible problems of financial asset reclassification.

## 3. Financial Data Intelligent Mining Classification Method

We assume that there are *p* indicators in the actual problem under discussion, and we regard these *p* indicators as *p* random variables, denoted as *X*_1_, *X*_2_,…, *X*_*p*_. Principal component analysis is to transform the problem of *p* indicators into a problem of discussing the linear combination of *p* indicators. These new indicators *F*_1_, *F*_2_,…, *F*_*k*_(*k* ≤ *p*) fully reflect the information of the original indicators in accordance with the principle of preserving the amount of main information and are independent of each other.

This process of reducing multiple indicators to a few comprehensive indicators is mathematically called dimensionality reduction. The usual method of principal component analysis is to seek the linear combination *F*_*i*_ of the original indicators.

(1) $$\begin{array}{c} F_{1}=\mu_{11}X_{1}+\mu_{21}X_{2}+\cdots \mu_{p1}X_{p},\\ F_{2}=\mu_{12}X_{1}+\mu_{22}X_{2}+\cdots \mu_{p2}X_{p},\\ \cdots \\ F_{p}=\mu_{1p}X_{1}+\mu_{2p}X_{2}+\cdots \mu_{pp}X_{p}. \end{array}$$

It satisfies the following conditions:

(1) The sum of the squares of the coefficients of each principal component is 1, that is,
  (2) $$\begin{array}{c} \mu_{1i}^{2}+\mu_{2i}^{2}+\cdots \mu_{pi}^{2}=1. \end{array}$$
(2) The principal components are independent of each other; that is, there is no overlapping information, that is,
  (3) $$\begin{array}{c} \text{Cov}\left(F_{i},F_{j}\right)=0,\;i\neq j,i,j=1,2,\ldots ,p. \end{array}$$
(3) The variance of the principal components decreases successively, and the importance decreases successively, namely,
  (4) $$\begin{array}{c} \text{Var}\left(F_{1}\right)\geq \text{Var}\left(F_{2}\right)\geq \cdots \geq \text{Var}\left(F_{p}\right). \end{array}$$

The conclusion of the two linear algebras is as follows:

(1) If *A* is a real symmetric matrix of order *p*, then an orthogonal matrix *U* must be found to make
  (5) $$\begin{array}{c} U^{-1}AU={\left[\begin{array}{cccc} \lambda_{1} & 0 & \cdots & 0\\ 0 & \lambda_{2} & \cdots & 0\\ \vdots & \vdots & \ddots & \vdots \\ 0 & 0 & \cdots & \lambda_{p} \end{array}\right]}_{p\times p}. \end{array}$$
Among them, *λ*_*i*_(*i*=1,2,…, *p*) is the characteristic root of *A*.
(2) If the unit eigenvector corresponding to the characteristic root of the above matrix is *μ*_1_,…, *μ*_*p*_,
  (6) $$\begin{array}{c} U=\left(u_{1},u_{2},\ldots ,u_{p}\right)=\left[\begin{array}{cccc} u_{11} & u_{12} & \cdots & u_{1p}\\ u_{21} & u_{22} & \cdots & u_{2p}\\ \vdots & \vdots & \ddots & \vdots \\ u_{p1} & u_{p2} & \cdots & u_{pp} \end{array}\right]. \end{array}$$

Then, the eigenvectors corresponding to different eigenvalues of the real symmetric matrix *U* are orthogonal, that is, *U*′*U*=*UU*′=*I* [22].

Conclusion: ∑*x* is the covariance matrix of the random vector *X*=*X*_1_+*X*_2_+*X*_3_+⋯+*X*_*p*_. It has eigenvalue *λ*_1_, *λ*_2_,…, *λ*_*p*_ and eigenvector *u*_1_, *u*_2_,…, *u*_*p*_, where *λ*_1_ ≥ *λ*_2_ ≥ ⋯≥*λ*_*p*_. Then, the principal components are

(7) $$\begin{array}{c} F_{1}=\mu_{11}X_{1}+\mu_{21}X_{2}+\cdots \mu_{p1}X_{p},\\ F_{2}=\mu_{12}X_{1}+\mu_{22}X_{2}+\cdots \mu_{p2}X_{p},\\ \cdots \\ F_{p}=\mu_{1p}X_{1}+\mu_{2p}X_{2}+\cdots \mu_{pp}X_{p}. \end{array}$$

At this time, Var(*F*_*i*_)=*U*′∑*xU*=*λ*_*i*_, *i* = 1, 2,…, *p*. Cov(*F*_*i*_, *F*_*j*_)=0, *i* ≠ *j*, *i*, *j*=1,2,…, *p*.

It is written in matrix form: *F*=*U*′*X*.

(8) $$\begin{array}{c} U=\left(u_{1},\ldots ,u_{p}\right)=\left[\begin{array}{cccc} u_{11} & u_{12} & \cdots & u_{1p}\\ u_{21} & u_{22} & \cdots & u_{2p}\\ \vdots & \vdots & \ddots & \vdots \\ u_{p1} & u_{p2} & \cdots & u_{pp} \end{array}\right],\\ X=\left(X_{1},X_{2},\ldots ,X_{P}\right). \end{array}$$

The two basic concepts are as follows:

1. Contribution rate: the proportion of the variance of the *i*-th principal component in the total variance, *λ*_*i*_/∑_*i*=1_^*p*^*λ*_*i*_, is called the contribution rate, which reflects how much information the original Р indicators have and how comprehensive they are.
2. Cumulative contribution rate: the comprehensive ability of the first *k*_*i*_ principal components is described by the variance of these *k* principal components and the proportion ∑_*i*=1_^*k*^*λ*_*i*_/∑_*i*=1_^*p*^*λ*_*i*_ in the total variance, which is called the cumulative contribution rate.

One of the purposes of our principal component bond analysis is to replace the original *p* indicators with as few principal components *F*_1_, *F*_2_,…, *F*_*k*_(*k* ≤ *p*) as possible. In actual work, the number of principal components depends on the amount of information that can reflect the original variable, that is, the cumulative contribution rate.

Principal component analysis is a method that converts multiple indicators into a few indicators and can maintain the correlation of the largest original data. The more important variance contribution *β*_*i*_(*i*=1,2,…, *k*) in principal component analysis represents the maximum value of the variance contribution of the *i*-th common factor after eliminating the influence of (*i*−1) common factors. It is mainly used to measure the importance of the *i*-th common factor. Therefore, the corresponding evaluation model can be established with *β* as the weight: *F*=*β*_1_*F*_1_+*β*_2_*F*_2_+⋯+*β*_*k*_*F*_*k*_. Among them, *F*_1_, *F*_2_,…, *F*_*k*_ is the corresponding *k* common factors used to comprehensively describe the original indicators, and the comprehensive score is calculated and sorted.

In summary, the calculation process of finding the principal components has the following steps. We set *n* samples, where each sample has *m* data, denoted as

(9) $$\begin{array}{c} X=\left[\begin{array}{ccc} x_{11} & \cdots & x_{1m}\\ \vdots & \ddots & \vdots \\ x_{n1} & \cdots & x_{nm} \end{array}\right]. \end{array}$$

(1) Standardization of sample data: in order to achieve the standardization of sample data, the mean and variance of the sample data need to be required. The standardization of sample data is based on the mean and variance of the data. The essence of standardization is to transform the sample into standardized data with a mean value of 0 and a variance of 1. That is, the normalized transformation of the column is
  (10) $$\begin{array}{c} x_{ij}^{•}=\frac{\left(x_{ij}-{\bar{x}}_{j}\right)}{\delta_{j}}. \end{array}$$
Among them,
  (11) $$\begin{array}{c} i=1,2,...,n,\\ j=1,2,...,m,\\ {\bar{x}}_{j}=\frac{1}{n}\sum_{i=1}^{n}x_{ij},\\ \delta_{j}^{2}=\frac{1}{n}\sum_{i=1}^{n}{\left(x_{ij}-{\bar{x}}_{j}\right)}^{2}. \end{array}$$
The resulting standardized matrix *X*^•^ is written as
  (12) $$\begin{array}{c} X=\left[\begin{array}{ccc} x_{11} & \cdots & x_{1m}\\ \vdots & \ddots & \vdots \\ x_{n1} & \cdots & x_{nm} \end{array}\right]. \end{array}$$
(2) Calculation of the correlation matrix: for given *n* samples, we use a computer to calculate the correlation coefficient matrix of the indicator variables:
  (13) $$\begin{array}{c} R=\left[\begin{array}{ccc} r_{11} & \cdots & r_{1m}\\ \vdots & \ddots & \vdots \\ r_{m1} & \cdots & r_{mm} \end{array}\right]=\frac{1}{n}X^{\prime }X. \end{array}$$
Among them,
  (14) $$\begin{array}{c} r_{ij}=\frac{1}{n}\sum_{i=1}^{n}X_{ij}X_{ik}=\frac{1}{n}x_{j}^{\prime }x_{k},\;j,k=1,2,\ldots ,m. \end{array}$$
(3) Find eigenvalues and eigenvectors. The obtained correlation matrix is *R* to solve the characteristic equation:
  (15) $$\begin{array}{c} \left|R-\lambda f\right|=0. \end{array}$$
By solving the characteristic equation, *k* eigenvalues (*i* = 1∼*m*) and the eigenvector corresponding to each eigenvalue can be obtained:
  (16) $$\begin{array}{c} Q_{i}=\left(a_{i1},a_{i2},\ldots ,a_{ip}\right),\;i=1∼k. \end{array}$$
And the eigenvectors corresponding to *λ*_1_ > *λ*_2_ > *λ*_3_ > *λ*_*k*_ > 0 are orthogonal to each other.
By the above method, *k* (*k* ≤ *p*) principal components can be obtained. *λ*_*i*_/∑_*i*=1_^*k*^*λ*_*i*_ is the contribution rate of the *i*-th principal component, denoted as *β*_*i*_, which is
  (17) $$\begin{array}{c} \beta_{i}=\frac{\lambda_{i}}{\sum_{i=1}^{k}\lambda_{i}}. \end{array}$$
Among the *k* principal components, the sum of the contribution rates of the first *q* principal components is the cumulative contribution rate of the first *q* principal components, denoted as *α*:
  (18) $$\begin{array}{c} \alpha =\frac{\sum_{i=1}^{q}\lambda_{i}}{\sum_{i=1}^{k}\lambda_{i}}. \end{array}$$
The number of principal components can be determined by the cumulative contribution rate. Usually the cumulative contribution rate *α* ≥ 0.85 as the standard. For the selected *q* principal components, if the cumulative contribution rate reaches 85%, that is, *a* ≥ 0.85, the principal components can be determined as *q*. It represents the selected *q* principal components and basically retains the information of the original *p* variables. When determining the number of principal components, the number of principal components should be minimized under the condition of the cumulative contribution rate constraint.
(4) Find the factor load *α*_*i*_:
  (19) $$\begin{array}{c} \alpha_{i}=\sqrt{\lambda_{i}}\alpha_{i}. \end{array}$$
According to formula (19), we calculate the factor loading matrix and then calculate the score of each factor:
  (20) $$\begin{array}{c} F_{i}=\alpha_{i}x,\;i=1,2,\ldots ,k. \end{array}$$
(5) According to the numerator of the factor and the size of the contribution rate, we calculate the comprehensive score:
  (21) $$\begin{array}{c} F=\beta_{1}F_{1}+\beta_{2}F_{2}+\cdots +\beta_{k}F_{k}. \end{array}$$
Finally, it is sorted according to the comprehensive score. PCA analyzes the system with fewer *q* indicators instead of the original *p* indicators, which brings great convenience to the comprehensive evaluation of the system.

A large amount of original detection value data flow information is used to detect lag correlation using the BRAID method, and a series of time series with lag correlation can be found, and the lag time of each time series relative to the original reference series can be calculated.

We assume that there are *n* time series with a lagging correlation after the detection of the BRAID method. Each time series has a piece of data. Since the original detection data are data values obtained at equal intervals of time, a time variable *t* is introduced in the representation of the time series, and the data value corresponding to each time point is *x* (*t*). Then the original multidimensional data flow is denoted as

(22) $$\begin{array}{c} X=\left(X_{1}\left(t\right),X_{2}\left(t\right),\ldots ,X_{n}\left(t\right)\right),\;t=0,1,\ldots ,m-1. \end{array}$$

This can be written in matrix form, namely,

(23) $$\begin{array}{c} X=\left[\begin{array}{cccc} x_{1}\left(0\right) & x_{1}\left(1\right) & \cdots & x_{1}\left(m-1\right)\\ x_{2}\left(0\right) & x_{2}\left(1\right) & \cdots & x_{2}\left(m-1\right)\\ \vdots & \vdots & \ddots & \vdots \\ x_{n}\left(0\right) & x_{n}\left(1\right) & \cdots & x_{n}\left(m-1\right) \end{array}\right]. \end{array}$$

The lag time is recorded as Δ*t*_1_, Δ*t*_2_,…, Δ*t*_*n*_.

Among them, Δ*t*_1_=0 (that is, *x* is the original reference sequence), and Δ*t*_2_,…, Δ*t*_*n*_ is the lag time of the sequence *x*_2_,…, *x*_*n*_ relative to the sequence *x*_1_.

**Figure 1:**
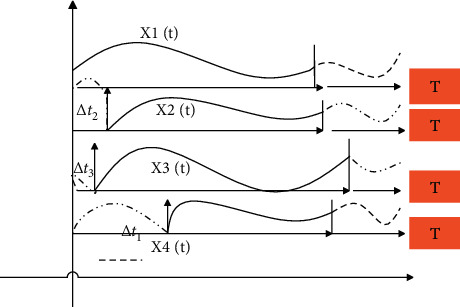
Unsynchronized time series.

**Figure 2:**
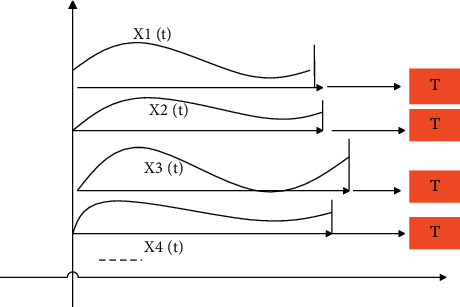
Synchronized time series.

The first principal component analysis method is applied to “aligned” data streams; that is, the data is synchronized. This article discusses the lag-related multidimensional data flow. The data that needs principal component analysis is not synchronized, but has a certain lag time relative to the original time series. Therefore, it is necessary to perform principal component analysis on the detected multidimensional data stream with lagging correlation. The first task is to remove the lagging part of each time series, and “align” the data flow, that is, temporarily ignore the time parameters, and only keep the parts with similar changing trends in each time series, in order to achieve the purpose of multidimensional data flow synchronization.  Figure 1 shows the unsynchronized time series, and Figure 2 shows the synchronized time series.

If the data corresponding to *w* time points in each data stream are obtained, the synchronized multidimensional data stream is recorded as

(24) $$\begin{array}{c} X=\left[\begin{array}{cccc} x_{1}\left(0\right) & x_{1}\left(1\right) & \cdots & x_{1}\left(w-1\right)\\ x_{2}\left(\Delta t_{2}\right) & x_{2}\left(\Delta t_{2}+1\right) & \cdots & x_{2}\left(\Delta t_{2}+w-1\right)\\ \vdots & \vdots & \ddots & \vdots \\ x_{i}\left(\Delta t_{i}\right) & x_{i}\left(\Delta t_{i}+i\right) & \cdots & x_{i}\left(\Delta t_{i}+w-1\right)\\ \vdots & \vdots & \ddots & \vdots \\ x_{n}\left(t_{n}\right) & x_{n}\left(\Delta t_{n}+i\right) & \cdots & x_{n}\left(\Delta t_{n}+w-1\right) \end{array}\right]. \end{array}$$

That is, *X*=(*X*_*i*_(Δ*t*_*i*_), *X*_*i*_(Δ*t*_*i*_+1),…, *X*_*i*_(Δ*t*_*i*_+*w* − 1)), where *i* = 1, 2,…, *n*.

For the above synchronized multidimensional data stream with delayed correlation, we apply the principal component analysis method to find the principal components. The steps of calculating the principal components include standardizing the sample data, calculating the correlation matrix, finding the eigenvalues and eigenvectors, and finding the principal components.

(1) Standardize multidimensional data streams. In order to achieve the standardization of multidimensional data streams, we should find the mean and variance of each data stream. The standardization of the data stream is based on the mean and variance of each data in the data stream. The essence of standardization is to transform the data stream into standardized data with a mean value of 0 and a variance of 1, which is to perform a standardized transformation on the column of *X*:
  (25) $$\begin{array}{c} x_{i}^{•}\left(j\right)=\frac{\left(x_{i}\left(j\right)-\bar{x\left(j\right)}\right)}{\delta_{j}}. \end{array}$$
Among them,
  (26) $$\begin{array}{c} i=1,2,\ldots ,n,\\ j=1,2,\ldots ,m,\\ \bar{x\left(j\right)}=\frac{1}{n}\sum_{i=1}^{n}x_{i}\left(j\right),\\ \delta_{j}^{2}=\frac{1}{n}\sum_{i=1}^{n}{\left(x_{i}\left(j\right)-\bar{x\left(j\right)}\right)}^{2}. \end{array}$$
Then we get the standardized matrix, denoted as
  (27) $$\begin{array}{c} X=\left[\begin{array}{cccc} x_{1}\left(0\right) & x_{1}\left(1\right) & \cdots & x_{1}\left(w-1\right)\\ x_{2}\left(0\right) & x_{2}\left(1\right) & \cdots & x_{2}\left(w-1\right)\\ \vdots & \vdots & \ddots & \vdots \\ x_{n}\left(0\right) & x_{n}\left(1\right) & \cdots & x_{n}\left(w-1\right) \end{array}\right]. \end{array}$$
(2) Calculate the correlation matrix. For the *w* data streams with lagging correlations that have been detected, we use a computer to calculate the correlation coefficient matrix of the indicator variables:
  (28) $$\begin{array}{c} R=\left[\begin{array}{ccc} r_{11} & \cdots & r_{1m}\\ \vdots & \ddots & \vdots \\ r_{m1} & \cdots & r_{mm} \end{array}\right]=\frac{1}{n}X^{\prime }X. \end{array}$$
Among them,
  (29) $$\begin{array}{c} r_{ij}=\frac{1}{n}\sum_{i=1}^{n}X_{ij}X_{ik}=\frac{1}{n}x_{j}^{\prime }x_{k},\;j,k=1,2,\ldots ,m. \end{array}$$
(3) Find eigenvalues and eigenvectors. The obtained correlation matrix is *R* to solve the characteristic equation: |*R* − *λf*|=0.
By solving the characteristic equation, *w* eigenvalues can be obtained, and the eigenvector corresponding to each eigenvalue: *U*_*i*_=(*u*_*i*1_, *u*_*i*2_,…, *u*_*iw*_), *i* = 1∼*w*, and *λ*_1_ > *λ*_2_ > ⋯>*λ*_*w*_ > 0, and its corresponding eigenvector are orthogonal to each other.
(4) Find the principal components. The algorithm can obtain *w* principal components by the above method. *λ*_*i*_/∑_*i*=1_^*w*^*λ*_*i*_ is the contribution rate of the *i*-th principal component, denoted as *β*_*i*_, namely,
  (30) $$\begin{array}{c} \beta_{i}=\frac{\lambda_{i}}{\sum_{i=1}^{w}\lambda_{i}}. \end{array}$$
Among the *w* principal components, the sum of the contribution rates of the first *k* principal components is called the cumulative contribution rate of the first *k* principal components, denoted as *α*:
  (31) $$\begin{array}{c} \alpha =\frac{\sum_{i=1}^{k}\lambda_{i}}{\sum_{i=1}^{w}\lambda_{1}}. \end{array}$$

The number of principal components can be determined by the cumulative contribution rate. This paper takes the cumulative contribution rate *α* ≥ 0.85 as the standard. For the selected *k* principal components, if the cumulative contribution rate reaches 85%, that is, *α* ≥ 0.85, then the principal components can be determined as *k*. It means that the selected *k* principal components basically retain the information of the original *w* variables. When determining the number of principal components, the number of principal components should be reduced as much as possible under the condition of *α* ≥ 0.85.

Using the obtained *k* principal components to linearly combine them can express the original multidimensional data flow of the lag correlation, namely,

(32) $$\begin{array}{c} F_{1}\left(t\right)=u_{11}x_{1}\left(t\right)+u_{12}x_{2}\left(t+\Delta t_{2}\right)+\cdots +u_{1i}x_{i}\left(t+\Delta t_{i}\right)+\cdots +u_{1k}x_{k}\left(t+\Delta t_{k}\right),\\ F_{2}\left(t\right)=u_{21}x_{1}\left(t-\Delta t_{2}\right)+u_{22}x_{2}\left(t\right)+\cdots +u_{2i}x_{i}\left(t+\Delta t_{i}-\Delta t_{2}\right)+\cdots +u_{2k}x_{k}\left(t+\Delta t_{k}-\Delta t_{2}\right),\\ \cdots \\ F_{i}\left(t\right)=u_{i1}x_{1}\left(t-\Delta t_{i}\right)+u_{i2}x_{2}\left(t-\Delta t_{i}+\Delta t_{2}\right)+\cdots +u_{ii}x_{i}\left(t\right)+\cdots +u_{ik}x_{k}\left(t-\Delta t_{i}-\Delta t_{k}\right),\\ \cdots \\ F_{n}\left(t\right)=u_{n1}x_{1}\left(t-\Delta t_{n}\right)+u_{n2}x_{2}\left(t-\Delta t_{n}+\Delta t_{2}\right)+\cdots +u_{ni}x_{i}\left(t-\Delta t_{n}+\Delta t_{i}\right)+\cdots +u_{nk}x_{k}\left(t+\Delta t_{n}-\Delta t_{k}\right). \end{array}$$

Furthermore, the algorithm obtains a comprehensive evaluation function:

(33) $$\begin{array}{c} F=\beta_{1}F_{1}+\beta_{2}F_{2}+\cdots +\beta_{k}F_{k}. \end{array}$$

It can be seen that, by performing principal component analysis (PCA) on the lag-related multidimensional data stream, the original multidimensional data stream represented by the data corresponding to the larger *m* time points can be converted into the smaller *k* principal components to represent the original multidimensional data stream, which brings great convenience to the storage and use of data stream and also provides help for the analysis and reconstruction of lag-related multidimensional data stream.

## 4. Classification and Statistics of Financial Data in Smart Cities Based on Artificial Intelligence

**Figure 3:**
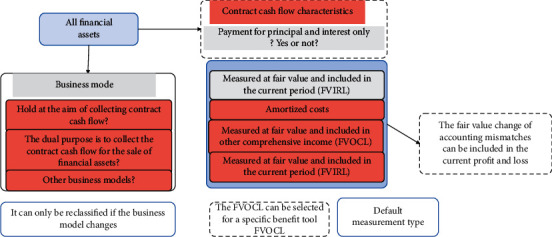
The overall framework of financial asset classification.

The overall framework of financial asset classification is shown in Figure 3.

**Figure 4:**
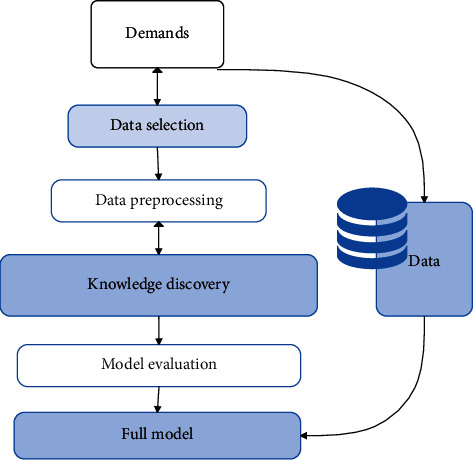
The data mining process.

There are three major factors in sequence analysis. (1) Sequence duration: it refers to the length of the analyzed sequence in the time dimension, such as the time period during which user behavior occurs. (2) Time folding window: it means that a series of actions occurring within a certain period of time will be regarded as occurring simultaneously. (3) Time interval: it refers to the time interval of the discovered time series model. An example of the data mining process is shown in Figure 4.

**Figure 5:**
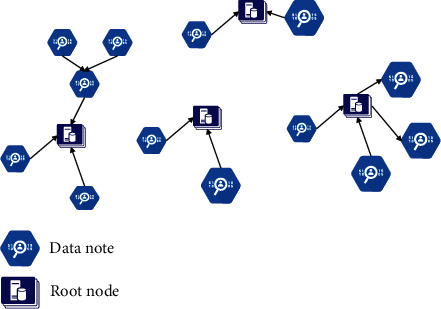
A possible result of finding the root node on the sample data set.

The fast hierarchical clustering algorithm based on root finding is based on the following assumption: a data set can be divided into several clusters, and there is a core point in each cluster, and this point can represent other points in the cluster. This core point is not necessarily the center of mass of the cluster; it exists in an area where the data is densely distributed. The area with dense data distribution here refers to the closest distance between the data points in this area relative to the surrounding neighborhood of this area. We call the core point of the cluster the root node, as shown in Figure 5.

**Figure 6:**
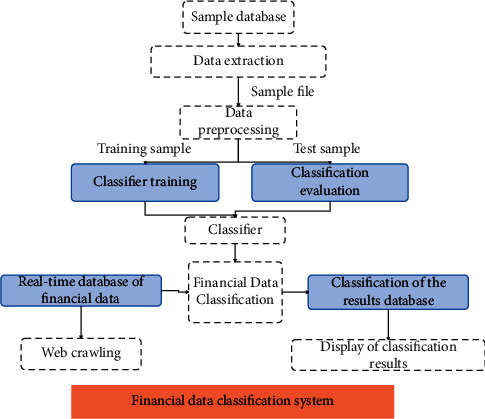
Financial data classification system structure.

The structure design of the financial data classification system is shown in Figure 6. The system obtains sample files from the sample database after data extraction and preprocesses the sample files into training samples and test samples. The training samples are used to train the classifier, and the test samples are used to evaluate the classification performance of the classifier, and the training process of the classifier is adjusted according to the evaluation results to obtain the final classifier.

**Figure 7:**
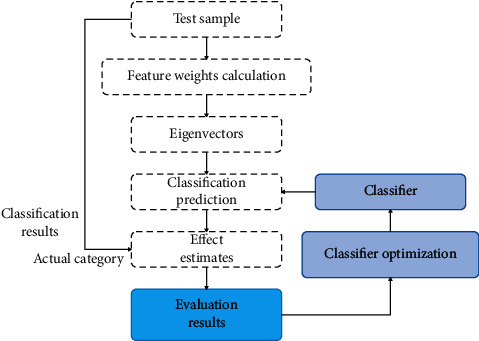
Classification and evaluation process diagram.

The evaluation process of the classification effect is shown in Figure 7. The source of the test sample is the same as the training sample. It is taken from the sample database and preprocessed. The test sample also carries the actual category label.

**Figure 8:**
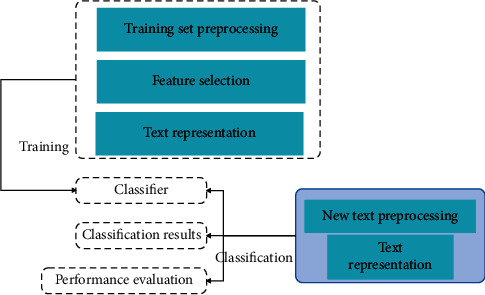
Principles of financial data classification.

The simple understanding of financial data classification task refers to the process of categorizing financial data of an unknown category based on its content when the classification system is known. Financial data classification is essentially a process of identifying the characteristics of financial data patterns. After the financial data is preprocessed and expressed as a feature vector, it is input into the classifier, and the model is trained. For the financial data to be classified, it is also expressed as a feature vector and then input into the classifier for classification.  Figure 8 shows the principle of financial data classification. Financial data content processing will be introduced in detail later.

**Figure 9:**
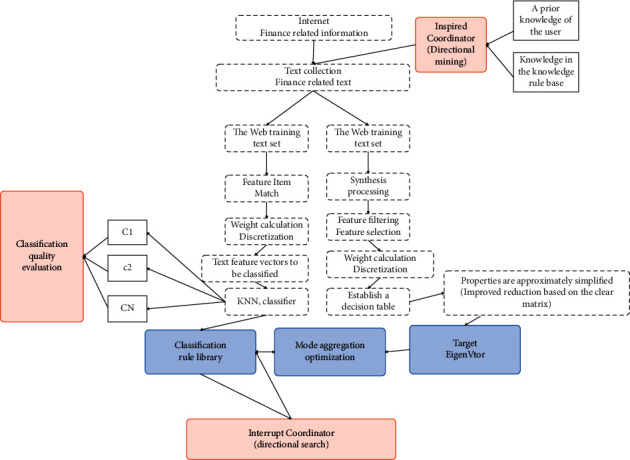
Financial data subsystem model framework.

As shown in Figure 9, we have given a basic model framework based on rough set and KNN text classification. In this model framework, we can clearly see that this model is composed of three parts: rough set-related preprocessing module, KNN classification algorithm module, and quality evaluation module. First, in the text collection stage, the relevant web text data is obtained from the internet, and the relevant data is used as the training set and test set of the web text for subsequent operations, and then the next step is entered.

**Figure 10:**
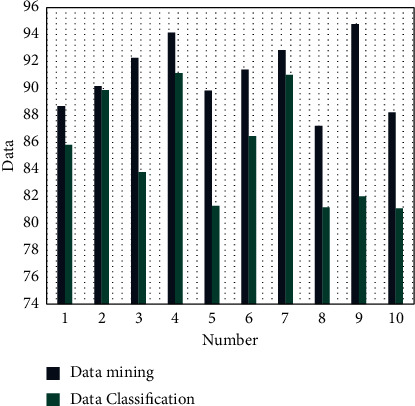
Evaluation of financial data processing effect.

After constructing the above intelligent model, this paper conducts statistical analysis of the intelligent model and evaluates the effect of financial data mining and financial data classification, and the results are shown in Figure 10.

**Figure 11:**
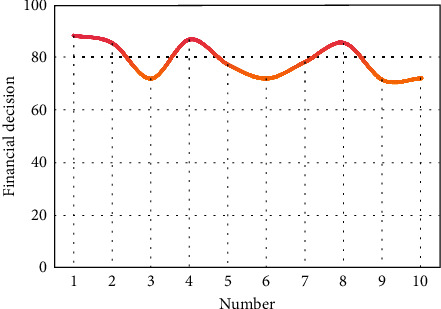
Intelligent data decision statistics.

It can be seen from Figure 10 that the intelligent algorithm proposed in this paper can play a certain role in financial data processing. After evaluating the intelligent data decision in this paper, the results are shown in Figure 11.

From the above research, we can see that the artificial intelligence classification and statistical method of financial data in smart cities proposed in this paper can play a certain auxiliary role in financial decision-making.

## 5. Conclusion

The prediction problem of financial data processing is mainly the prediction of financial time series. Financial time series can be regarded as a special time series, which has the following three characteristics. (1) The generation process of financial time series is more complicated, and there are many influencing factors. (2) Most financial time series contain a large number of unpredictable influence factors. (3) The composition of the data in the financial time series is relatively complex and usually exhibits nonlinearity. Artificial intelligence information processing methods such as neural networks, chaos theory, and genetic algorithms can well adapt to these three characteristics, which have become advanced methods to solve financial data processing problems. This paper combines big data technology to carry out financial data classification research and extracts financial data from different data sources in an automatic and intelligent way. Users can not only get the data they want in the shortest time, but also improve the accuracy and effectiveness of the data. Through experimental research, we know that the artificial intelligence classification and statistical method of financial data in smart cities proposed in this paper can play a certain auxiliary role in financial decision-making.

## Data Availability

The labeled dataset used to support the findings of this study is available from the corresponding author upon request.
